# Supplementary material for: Emergence of multifrequency activity in a laminar neural mass model
Source: PLoS Comput Biol. 2026 Apr 3;22(4):e1014022. doi: 10.1371/journal.pcbi.1014022 (PMC13075798; doi:10.1371/journal.pcbi.1014022)
Supplement: S1 Material — (PDF) [file pcbi.1014022.s001.pdf]

Supplementary material for: Emergence of multifrequency activity in a laminar neural mass model

Raul de Palma Aristides[1], Pau Clusella[2], Roser Sanchez-Todo[3,4], Giulio Ruffini[4], and Jordi Garcia-Ojalvo[1]

[1]Department of Medicine and Life Sciences, Universitat Pompeu Fabra, Barcelona, Spain
[2]Department of Mathematics, Universitat Politècnica de Catalunya, Manresa, Spain
[3]Center of Brain and Cognition, Universitat Pompeu Fabra, Barcelona, Spain
[4]Brain Modeling Department, Neuroelectrics, Barcelona, Spain

October 10, 2025

1 Supplementary material

All code used to implement the methods described below is available at https://github.com/dsb-lab/LaNMM.

1.1 Single column model

Following the diagram shown in Fig. 1 of the main text, the LaNMM is given in the synapse-driven formalism by [1]

$$\hat{L}_s[u_i(t)] = \frac{1}{A_s}\left(\frac{1}{a_s}\frac{d^2}{dt^2} + 2\frac{d}{dt} + a_s\right)u_i(t)\ . \tag{1}$$

with

$$
\begin{aligned}
\hat{L}[u_1(t)] &= C_1\sigma_{SS}(v_{SS}) \\
\hat{L}[u_2(t)] &= C_2\sigma_{SST}(v_{SST}) \\
\hat{L}[u_3(t)] &= C_3(\varphi_{e1}) \\
\hat{L}[u_4(t)] &= C_4\sigma_{P_1}(v_{P_1}) \\
\hat{L}[u_5(t)] &= C_5\sigma_{P_1}(v_{P_1}) \\
\hat{L}[u_6(t)] &= C_6\sigma_{P_2}(v_{P_2}) \\
\hat{L}[u_7(t)] &= C_7\sigma_{PV}(v_{PV}) \\
\hat{L}[u_8(t)] &= C_8(\varphi_{e2}) \\
\hat{L}[u_9(t)] &= C_9\sigma_{P_2}(v_{P_2}) \\
\hat{L}[u_{10}(t)] &= C_{10}\sigma_{PV}(v_{PV}) \\
\hat{L}[u_{11}(t)] &= C_{11}\sigma_{P_2}(v_{P_2}) \\
\hat{L}[u_{12}(t)] &= C_{12}\sigma_{P_1}(v_{P_1}) \\
\hat{L}[u_{13}(t)] &= C_{13}\sigma_{P_1}(v_{P_1})
\end{aligned}
$$

Where s is the type of neurotransmitter and i the population in question. Notice that $\hat{L}[u(t)]$ has a dimension of frequency (Hz). The average membrane potentials are given by:

$$
\begin{aligned}
v_{P_1} &= u_1 + u_2 + u_3 + u_{11} && (2) \\
v_{SS} &= u_4 && (3) \\
v_{SST} &= u_5 && (4) \\
v_{P_2} &= u_6 + u_7 + u_8 + u_{12} && (5) \\
v_{PV} &= u_9 + u_{10} + u_{13} && (6)
\end{aligned}
$$

Using the above equations and taking out the repeated synapses, and since all the synapses coming from one population have the same dynamics and the only difference is the coupling strength relative to the targeting population, we get

$$
\begin{aligned}
\hat{L}[u_1(t)] &= C_1\sigma_{SS}(u_4) && (7) \\
\hat{L}[u_2(t)] &= C_2\sigma_{SST}(u_5) && (8) \\
\hat{L}[u_3(t)] &= C_3(\varphi_{e1}) && (9) \\
\hat{L}[u_4(t)] &= C_4\sigma_{P_1}(u_1 + u_2 + u_3 + u_{11}) && (10) \\
\hat{L}[u_6(t)] &= C_6\sigma_{P_2}(u_6 + u_7 + u_8 + u_{12}) && (11) \\
\hat{L}[u_7(t)] &= C_7\sigma_{PV}(u_9 + u_{10} + u_{13}) && (12) \\
\hat{L}[u_8(t)] &= C_8(\varphi_{e2}) \ . && (13)
\end{aligned}
$$

Notice that u_4, u_5, u_{12}, and u_{13} all represent the perturbation caused by P_1, which will be weighted by the average number of connections between P_1 and the targets, u_4. Similarly,

$u_6 = u_9 = u_{11}$ and $u_7 = u_{10}$. Using these identities we can write:

$$\hat{L}[u_1(t)] = C_1\sigma_{SS}(u_4) \tag{14}$$
$$\hat{L}[u_2(t)] = C_2\sigma_{SST}(u_4) \tag{15}$$
$$\hat{L}[u_3(t)] = C_3(\varphi_{e1}) \tag{16}$$
$$\hat{L}[u_4(t)] = C_4\sigma_{P_1}(u_1 + u_2 + u_3 + u_6) \tag{17}$$
$$\hat{L}[u_6(t)] = C_6\sigma_{P_2}(u_6 + u_7 + u_8 + u_4) \tag{18}$$
$$\hat{L}[u_7(t)] = C_7\sigma_{PV}(u_6 + u_7 + u_4) \tag{19}$$
$$\hat{L}[u_8(t)] = C_8(\varphi_{e2}) \ . \tag{20}$$

For the sake of coherence with the main text we will introduce the change of variables:

$$u_4 \to u_1 \tag{21}$$
$$u_1 \to u_2 \tag{22}$$
$$u_2 \to u_3 \tag{23}$$
$$u_3 \to u_{\varphi_{e1}} \tag{24}$$
$$u_6 \to u_4 \tag{25}$$
$$u_7 \to u_5 \tag{26}$$
$$u_8 \to u_{\varphi_{e2}} \tag{27}$$

which gives us:

$$\hat{L}[u_2(t)] = C_1\sigma_{SS}(u_1) \tag{28}$$
$$\hat{L}[u_3(t)] = C_2\sigma_{SST}(u_1) \tag{29}$$
$$\hat{L}[u_{\varphi_{e1}}(t)] = C_3(\varphi_{e1}) \tag{30}$$
$$\hat{L}[u_1(t)] = C_4\sigma_{P_1}(u_2 + u_3 + u_{\varphi 1} + u_1) \tag{31}$$
$$\hat{L}[u_6(t)] = C_4\sigma_{P_2}(u_4 + u_5 + u_{\varphi 2} + u_1) \tag{32}$$
$$\hat{L}[u_7(t)] = C_5\sigma_{PV}(u_4 + u_5 + u_1) \tag{33}$$
$$\hat{L}[u_{\varphi_{e2}}(t)] = C_8(\varphi_{e2}) \ . \tag{34}$$

We now introduce a change of variables $u_i = C_j y_i$, where C_j accounts for the connectivity of the incoming synapse. The first equation, represents the process of receiving an average synapse and producing a membrane perturbation from the *point of view* of the SST population. The incoming average synapse from P_1 is weighted by C_4 and the outgoing average synapse to P_1 is weighted by C_1. Taking this into account we have

$$\begin{aligned}
\hat{L}_4[u_2(t)] &= C_1\sigma_{SS}(u_1) \\
\frac{1}{C_1}\hat{L}_1[u_2(t)] &= \sigma_{SS}(C_4 y_1) \\
\hat{L}_1\Big[\frac{u_2(t)}{C_1}\Big] &= \sigma_{SS}(C_4 y_1) \\
\hat{L}_1[y_2(t)] &= \sigma_{SS}(C_4 y_1) \\
\frac{1}{A_1}\Big(\frac{1}{a_1}\frac{d^2}{dt^2} + 2\frac{d}{dt} + a_1\Big)[y_2(t)] &= \sigma_{SS}(C_4 y_1) \\
\ddot{y_2} &= a_1 A_1 \sigma_{SS}(C_4 y_1(t)) - 2a_1\dot{y}_2(t) - a_1^2 y_2(t) \ .
\end{aligned}$$

For the external inputs, we have

$$\hat{L}_3[u_{\varphi 1}(t)] = C_3(\varphi_{e1}) \tag{35}$$

$$\frac{1}{A_3}\Big(\frac{1}{a_3}\frac{d^2}{dt^2} - 2\frac{d}{dt} + a_3\Big)[y_{\varphi_{e1}}(t)] = \varphi_{e1}, \quad \text{and if } y_{\varphi_{e1}} \text{ does not depend on time} \tag{36}$$

$$y_{\varphi_{e1}} = \frac{A_3}{a_3}\varphi_{e1}. \tag{37}$$

Similarly, for s_8 we have $y_{\varphi_{e1}} = \dfrac{A_8}{a_8}\varphi_{e2}$. Putting these results together and using the correspondent parameters for A's and a's we have:

$$\ddot{y}_1 = a_A A_A \sigma_{P_1}(C_1 y_2 + C_2 y_3 + C_3\varphi_{e1} + C_{11} y_4) - 2a_A \dot{y}_1 - a_A^2 y_1 \tag{38a}$$
$$\ddot{y}_2 = a_A A_A \sigma_{SS}(C_4 y_1) - 2a_A \dot{y}_2 - a_A^2 y_2 \tag{38b}$$
$$\ddot{y}_3 = a_{G_s} A_{G_s} \sigma_{SST}(C_5 y_1) - 2a_{G_s} \dot{y}_3 - a_{G_s}^2 y_3 \tag{38c}$$
$$\ddot{y}_4 = a_A A_A \sigma_{P_2}(C_6 y_4 + C_7 y_5 + C_8\varphi_{e2} + C_{12} y_1) - 2a_A \dot{y}_4 - a_A^2 y_4 \tag{38d}$$
$$\ddot{y}_5 = a_{G_f} A_{G_f} \sigma_{PV}(C_9 y_4 + C_{10} y_5 + C_{13} y_1) - 2a_{G_f} \dot{y}_5 - a_{G_f}^2 y_5 \tag{38e}$$

where each one of these second-order equations can be rewritten as a system of two first-order equations using $\dot{y}_i(t) = z_i(t)$.

1.2 Two-column model

Following the diagram shown in Fig. 11 the equations of the two column model are given by:

$$\ddot{y}_{11} = a_A A_A \sigma_{P_1}(C_1 y_{12} + C_2 y_{13} + C_3\varphi + C_{11} y_{14} + \kappa C_{14} y_{21}) - 2a_A \dot{y}_{11} - a_A^2 y_{11} \tag{39a}$$
$$\dot{y}_{12} = a_A A_A \sigma_{SS}(C_4 y_{11}) - 2a_A \dot{y}_{12} - a_A^2 y_{12} \tag{39b}$$
$$\ddot{y}_{13} = a_{G_s} A_{G_s} \sigma_{SST}[\kappa C_5(y_{11} + y_{21})] - 2a_{G_s} \dot{y}_{13} - a_{G_s}^2 y_{13} \tag{39c}$$
$$\dot{y}_{14} = a_A A_A \sigma_{P_2}(C_6 y_{14} + C_7 y_{15} + C_8\varphi/2 + \kappa C_{12}(y_{11} + y_{21})) - 2a_A \dot{y}_{14} - a_A^2 y_{14} \tag{39d}$$
$$\ddot{y}_{15} = a_{G_f} A_{G_f} \sigma_{PV}(C_9 y_{14} + C_{10} y_{15} + C_{13} y_1) - 2a_{G_f} \dot{y}_{15} - a_{G_f}^2 y_{15} \tag{39e}$$
$$\ddot{y}_{21} = a_A A_A \sigma_{P_1}(C_1 y_{22} + C_2 y_{23} + C_3\varphi + C_{21} y_4) - 2a_A \dot{y}_{21} - a_A^2 y_{21} \tag{39f}$$
$$\ddot{y}_{22} = a_A A_A \sigma_{SS}(C_4 y_{21}) - 2a_A \dot{y}_{22} - a_A^2 y_{22} \tag{39g}$$
$$\ddot{y}_{23} = a_{G_s} A_{G_s} \sigma_{SST}(C_5 y_{21}) - 2a_{G_s} \dot{y}_{23} - a_{G_s}^2 y_{23} \tag{39h}$$
$$\ddot{y}_{24} = a_A A_A \sigma_{P_2}[\kappa C_6(y_{14} + y_{24}) + C_7 y_{25} + C_8\varphi/2 + C_{12} y_{21}] - 2a_A \dot{y}_{24} - a_A^2 y_{24} \tag{39i}$$
$$\ddot{y}_{25} = a_{G_f} A_{G_f} \sigma_{PV}(C_9 y_{24} + C_{10} y_{25} + C_{13} y_{21}) - 2a_{G_f} \dot{y}_{25} - a_{G_f}^2 y_{25} \tag{39j}$$

To balance the influence of additional inputs on the populations, we introduce the parameter κ, which controls the strength of these inputs. For instance, in Eq. (39c), the SST_{11} population receives excitatory inputs from both P_{11} and P_{21}, and κ modulates the contribution of these inputs. Additionally, we introduce C_{14}, which represents the number of synaptic connections between the P_{21} and P_{11} populations. All numerical simulations and bifurcation diagrams were performed with $\kappa = 0.6$ and $C_{14} = 56.25$. Note that $\kappa = 0.0$ corresponds to the case where the two columns are uncoupled. The impact of this parameter on the system's dynamics will be explored in future work.

1.3 Numerical simulation and frequency analysis

Equations (38) were numerically solved using Python's `scipy.solve_ivp` function [2], employing a fourth-order Runge-Kutta algorithm, over a 100-second duration after discarding a 10-second transient, with an integration step of $dt = 0.001$. The frequency analysis for Fig. 4 was performed using a multitaper method implemented with `scipy.fft.fft` [2], using 1.25s windows (1250 samples), 50% overlap, and 5 Slepian tapers ($NW = 2.5$). For computational efficiency, in Fig. 7, the entire signal was used with windowing and padding, and the peak frequency was obtained for P_1 and P_2.

1.4 Bifurcation diagrams

All bifurcation diagrams were calculated with AUTO-07p, a publicly available software for continuation and bifurcation problems [3].

1.5 Lyapunov exponents

Lyapunov exponents are commonly used to assess how perturbations affect the trajectory of a system over time. A system has as many Lyapunov exponents as its dimensions, but by focusing on the two largest, λ_1 and λ_2, we can classify the system's dynamics as follows:

1. Fixed points: both exponents negative ($\lambda_1, \lambda_2 < 0$).
2. Periodic dynamics: one zero exponent, the rest negative ($\lambda_1 = 0, \lambda_2 < 0$).
3. Quasiperiodic dynamics: both exponents zero ($\lambda_1 = 0, \lambda_2 = 0$).
4. Chaotic dynamics: one positive exponent ($\lambda_1 > 0, \lambda_2 \leq 0$).

The Lyapunov exponents for Eq. (38) were calculated using the `lyapunovspectrum` function from the ChaosTools module in Julia, part of the DynamicalSystems library [4, 5]. A threshold of $|\lambda_k| < 10^{-3}$ was applied to differentiate between zero and non-zero values.
